# Biobutanol production from underutilized substrates using *Clostridium*: Unlocking untapped potential for sustainable energy development

**DOI:** 10.1016/j.crmicr.2024.100250

**Published:** 2024-06-08

**Authors:** Devina Syifa Nabila, Rosamond Chan, Rizky Riscahya Pratama Syamsuri, Puspita Nurlilasari, Wan Abd Al Qadr Imad Wan-Mohtar, Abdullah Bilal Ozturk, Nia Rossiana, Febri Doni

**Affiliations:** a Department of Biology, Faculty of Mathematics and Natural Sciences, Universitas Padjadjaran, Jatinangor, West Java 45363, Indonesia; b Doctorate Program in Biotechnology, Graduate School, Universitas Padjadjaran, Bandung, West Java 40132, Indonesia; c Department of Agro-industrial Technology, Faculty of Agro-industrial Technology, Universitas Padjadjaran, Jatinangor, West Java 45363, Indonesia; d Functional Omics and Bioprocess Development Laboratory, Institute of Biological Sciences, Faculty of Science, Universiti Malaya, Kuala Lumpur 50603, Malaysia; e Department of Chemical Engineering, Faculty of Chemical and Metallurgical Engineering, Yildiz Technical University, Esenler, Istanbul 34220, Türkiye

**Keywords:** Biobutanol, Biofuels, *Clostridium*, Green economy, Sustainable energy, Underutilized substrates

## Abstract

The increasing demand for sustainable energy has brought biobutanol as a potential substitute for fossil fuels. The *Clostridium* genus is deemed essential for biobutanol synthesis due to its capability to utilize various substrates. However, challenges in maintaining fermentation continuity and achieving commercialization persist due to existing barriers, including butanol toxicity to *Clostridium*, low substrate utilization rates, and high production costs. Proper substrate selection significantly impacts fermentation efficiency, final product quality, and economic feasibility in *Clostridium* biobutanol production. This review examines underutilized substrates for biobutanol production by *Clostridium,* which offer opportunities for environmental sustainability and a green economy. Extensive research on *Clostridium*, focusing on strain development and genetic engineering, is essential to enhance biobutanol production. Additionally, critical suggestions for optimizing substrate selection to enhance *Clostridium* biobutanol production efficiency are also provided in this review. In the future, cost reduction and advancements in biotechnology may make biobutanol a viable alternative to fossil fuels.

## Highlights

• *Clostridium* shows promise in synthesizing biobutanol, a potential alternative to fossil fuels.
• *Clostridium* can utilize diverse substrates such as organic waste for producing biobutanol.
• Challenges in commercializing *Clostridium*-based biobutanol production include butanol toxicity, slow growth, and high costs.
• Optimizing underutilized substrates in biobutanol synthesis with *clostridium* supports SDG7.

## Graphical abstract

**Figure:**
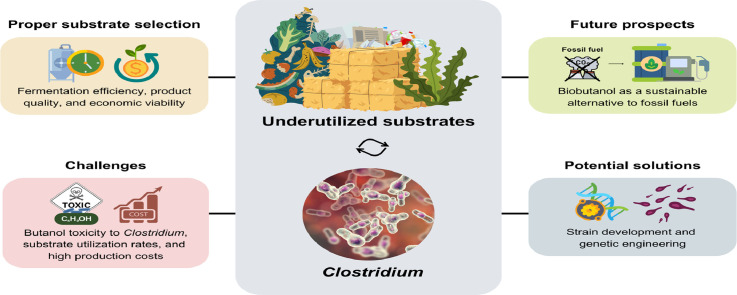

## 1 Introduction

The anticipated global population growth, projected to surpass 9 billion by 2050, has significant implications for worldwide energy demands and the urgent need for waste reduction (Neupane, 2023; Palaniswamy et al., 2023). Current data underscores the dominant role of fossil fuels, which presently account for 83 % of global energy requirements (Pugazhendhi et al., 2019). The extensive use of fossil fuels leads to a significant energy crisis, thereby seriously impacting fuel prices and the operations of various industries (Nandhini et al., 2023).

Furthermore, it is crucial to acknowledge that beyond the issue of oil reservoir depletion, the use of fossil fuels also poses profound environmental consequences (Neupane, 2023). Fossil fuels prominently exacerbate the escalation of greenhouse gas (GHG) emissions, notably carbon dioxide (CO_2_) (Pugazhendhi et al., 2019; Neupane, 2023). Therefore, substituting fossil fuels with environmentally friendly alternatives becomes paramount, prompting the world to officially incorporate efforts to achieve renewable energy targets aligned with Sustainable Development Goals (SDGs), particularly Goal number 7, "Affordable and Clean Energy'' (He et al., 2022a). Several objectives within SDG7 are outlined as follows: (1) markedly increasing the proportion of renewable energy in the worldwide energy mix by 2030; (2) doubling the pace of enhancement in global energy efficiency; and (3) boosting the provision of modern and sustainable energy services for all developing countries through infrastructure expansion and technological enhancement (Acheampong et al., 2017; Gebara and Laurent, 2023). This has led to a high interest in the search for sustainable energy sources in recent years, thus driving exploration focused on alternative energies such as biofuels (Acheampong et al., 2017; Adewuyi, 2020; Trinh and Chung, 2023).

Renewable energy sources play a crucial role in reducing the adverse impacts of carbon emissions and other environmental issues (Zafar et al., 2020; Al-Shetwi, 2022). The main advantages of renewable energy include climate change mitigation, cost reduction, and resilience to fluctuating prices (He et al., 2022a). Biobutanol is regarded as an exceptionally suitable sustainable biofuel option due to its superior fuel properties, which include high energy content, low volatility, low hygroscopicity, elevated energy density, a less corrosive nature, and the ability to blend with gasoline at ratios of up to 85 % (Gottumukkala et al., 2019; Tsai et al., 2020). Moreover, biobutanol can replace gasoline in internal combustion engines without requiring any modifications (Ndaba et al., 2015).

The main bacteria capable of producing biobutanol include the genus *Clostridium* (Liberato et al., 2019; Lin et al., 2023). Biobutanol is produced by *Clostridium* through fermentation utilizing both simple and complex sugar substrates, encompassing pentoses and hexoses (Jang et al., 2012), as well as various organic substrates, such as agricultural residues, industrial waste, and C1 gases (Huzir et al., 2018; Yang et al., 2022). That renders it an appealing option for sustainability and resource diversification (Huzir et al., 2018; Yang et al., 2022).

In biobutanol production, the substrate type, composition, and concentration are crucial factors that significantly impact productivity, yield, and efficiency (Visioli et al., 2014; Huzir et al., 2018). It is reported that the appropriate substrate concentration can enhance fermentation efficiency significantly while avoiding the waste of raw materials (Amin et al., 2024). However, raw material expenses account for over 70 % of production costs, making substrate selection a critical factor in achieving economic competitiveness (Visioli et al., 2014). A promising strategy that can be employed to reduce the production costs of biobutanol is to use inexpensive and renewable raw materials, such as agricultural waste, municipal wastes, and other sustainable sources like algae (Cheng et al., 2012; Palaniswamy et al., 2023; Cavelius et al., 2023).

The waste sector emerges as a significant contributor, accounting for 5 % of global GHG emissions (Kristanto et al., 2020). A substantial volume of biomass waste is generated annually worldwide, with rice straw totaling approximately 731.3 million tons, wheat straw up to 354.34 million tons, sugarcane bagasse amounting to 180.73 million tons, and corn stover reaching 128.02 million tons, being the most abundantly produced (Kalak et al., 2023). Therefore, it is crucial to focus on the proper management of organic waste (Akın et al., 2023).

Furthermore, the decomposition process of waste, especially agricultural waste, takes years if not managed properly, resulting in environmental pollution and energy resource loss. Therefore, the reutilization of residues through biotechnological recycling technology becomes a crucial step in maintaining resource sustainability (Zahroh et al., 2021; Tagne et al., 2022). Additionally, the use of microalgae as a raw material for biobutanol is not in competition with land use for agriculture or food production (Yeong et al., 2018). Microalgae biomass contains carbohydrates that can be utilized as a carbon source in the fermentation process for biobutanol production (Shanmugam et al., 2021; Khan et al., 2023; Yang et al., 2023). Moreover, syngas fermentation turns waste into gas with an energy efficiency ranging from 75 to 80 %, contingent on the composition and moisture content of the raw materials, resulting in a total plant efficiency of 57 %, thus making it economically feasible for biomass conversion (Dhakal and Acharya, 2021).

This conversion of organic waste into biofuel offers several positive impacts, including promoting sustainable energy resources, mitigating adverse environmental impacts, and reducing greenhouse gas emissions (Akın et al., 2023). By utilizing abundant organic waste resources for butanol and energy production, this approach addresses the need for affordable and clean energy, thus contributing to achieving the objectives of SDG7 (Acheampong et al., 2017; Nazari et al., 2020; Khan et al., 2021).

This article comprehensively discusses the production of biobutanol from bacteria of the genus *Clostridium*, highlighting key benefits such as the utilization of various substrates as renewable energy sources, cost comparison, state-of-the-art biotechnology application methods, and its advantages and limitations. Furthermore, it brings innovation to the field of biotechnology by exploring the use of underutilized substrates, such as agricultural waste and microalgae, which have been less extensively studied in the literature. It also emphasizes the knowledge gap in the specific application of *Clostridium* in the context of biomass valorization and alternative residue conversion.

The fermentation process with commonly used substrates, such as waste, is often hindered by multiple factors, including the presence of inhibitory substances (Mahapatra and Kumar, 2017; Abo et al., 2019). These constraints impede the efficient utilization of carbon substrates by *Clostridium* during fermentation (Abo et al., 2019). Closing this knowledge gap is crucial for fully uncovering the potential of biobutanol production using *Clostridium* and advancing environmentally friendly biofuel technology. This review aims to enhance understanding of the current landscape, challenges faced, and opportunities surrounding the use of *Clostridium* species for biobutanol production from underutilized substrates. By providing a comprehensive evaluation, this review lays the groundwork for developing more efficient and sustainable biobutanol production methods. It is anticipated that biobutanol will play a significant role in addressing the global energy and environmental challenges faced by today's interconnected world.

## 2 *Clostridium* species as biobutanol producers

*Clostridium* is a primary bacterial species known for its natural butanol production and has been extensively developed in industrial settings (Moon et al., 2016; Lin et al., 2023). Numerous vital fermentation processes, including the production of acetone and ethanol (Sreekumar et al., 2015), lactic acid (Mattiello-Franisco et al., 2021), butyric acid and acetic acid (Linger et al., 2020; Cao et al., 2022; Liberato et al., 2019), as well as succinic acid (Phuengjayaem et al., 2020), are facilitated by the genus *Clostridium. Clostridium* species produce various cellulolytic and hemicellulolytic enzymes that enable them to efficiently break down the complex structure of lignocellulosic biomass (Chukwuma et al., 2021; Dharmaraja et al., 2020; Palaniswamy et al., 2023). These enzymes, such as amylases, glucosidases, cellulases, hemicellulases, and lignases, hydrolyze components into simpler sugars (Vélez-Mercado et al., 2021; Tekin et al., 2023). A diverse array of carbon substrates, encompassing glucose, galactose, mannose, xylose, cellobiose, arabinose and complex organic compounds like lignocellulosic waste are utilized by *Clostridium* for the synthesis of carboxylic acids (acetate and butyrate) and solvents such as acetone, butanol, and ethanol (ABE) via glycolysis and the non-oxidative pentose phosphate pathway (Jones et al., 2023; Ezeji and Blaschek, 2008; Rathour et al., 2018; Mujtaba et al., 2023).

*Clostridium* is a type of Gram-positive bacterium characterized by its rod-shaped form, anaerobic nature, ability to form endospores, incapacity for sulfate assimilation reduction (Berezina et al., 2012; Janssen et al., 2014; Liberato et al., 2019), mesophilic nature, and being equipped with peritrichous flagella (Figueiredo et al., 2020; Liberato et al., 2019). The exclusive ability to produce biobutanol is confined to specific *Clostridium* bacterial species, including *C. acetobutylicum, C. saccharobutylicum, C. thermocellum, C. beijerinckii, C. saccharoperbutylacetonicum, C. aurantibutyricum, C. pasteurianum, C. sporogenes, C. carboxidivorans, C. tetanomorphum, C. aurantibutyricum,* and *C. kadaveris* (Moon et al., 2016; Iyyappan, 2021; Lin et al., 2023). Commonly, strains producing butanol are categorized into two groups based on their substrate utilization characteristics: those metabolizing starch, exemplified by *C. acetobutylicum* ATCC 824 and *C. acetobutylicum* DSM 1731, and those metabolizing sugars, such as *C. acetobutylicum* NCIMB8052, *C. acetobutylicum* P262, and *C. beijerinckii* BA101 (Lin et al., 2023). Among the microorganisms that produce butanol, *C. acetobutylicum, C. beijerinckii, C. sacharoperbutylacetonicum,* and *C. sacharoacetobutylicum* are commonly utilized in industry because they produce relatively high levels of butanol under favorable conditions (Li et al., 2020).

*Clostridium* species produce biobutanol through ABE fermentation, a dual-phase process that converts sugars into carboxylic acids (such as acetate and butyrate) and solvents (like acetone, butanol, and ethanol) (Buehler and Mesbah, 2016; Karstens et al., 2021; Lin et al., 2023). The initial acidogenic phase produces carboxylic acids, and this accumulation leads to a pH decrease in the medium (Niglio et al., 2019; Quintero-Díaz et al., 2023). The metabolism re-assimilates the acids to produce solvents as it transitions to the solventogenic phase (Liao et al., 2015; Guo et al., 2022; Lin et al., 2023). In the solventogenic phase, acetyl-CoA, butyryl-CoA, and acetoacetyl-CoA serve as precursors for ethanol, butanol, and acetone synthesis, respectively, being the three major intermediate metabolites in the ABE synthesis pathway (Lin et al., 2023). Acetone, butanol, and ethanol are produced in a ratio of 3:6:1, respectively (Quintero-Díaz et al., 2023).

*Clostridium* can also make butanol through two different fermentation processes: the isopropanol-butanol-ethanol (IBE) pathway and the hexanol-butanol-ethanol (HBE) pathway. These processes use different types of sugars and syngas from burning biomass (Tomita et al., 2019; Zhang et al., 2018a; Vieira et al., 2022; Fernández-Naveira et al., 2017b; Liberato et al., 2019; Benevenuti et al., 2020). The IBE fermentation process involves three main stages: the formation of acetic acid and butyrate during exponential growth, transitioning to neutral solvent formation as fermentation progresses, and the production of isopropanol, with timing varying among bacterial strains (Collas et al., 2012; Xin et al., 2018). The hexanol-butanol-ethanol (HBE) fermentation pathway involves the synthesis of a bioalcohol mixture from C1 gas, derived from biomass gasification known as syngas (Fernández-Blanco et al., 2022). This process combines thermochemical and biochemical processes to convert synthesis gas into ethanol and higher alcohols such as butanol and hexanol (Liberato et al., 2019). Organisms such as *C. ljungdahlii, C. carboxidivorans, C. autoethanogenum,* and *C. ragsdale* utilize the Wood-Ljungdahl pathway for acetyl-CoA synthesis, energy conservation, and alcohol production (Benevenuti et al., 2020). Overall, the primary distinctions among these three pathways are influenced by the substrate used and the variety of *Clostridium* involved in sustainable biofuel production and biotechnological applications.

## 3 Fermentation feedstocks

The choice of appropriate feedstock is a pivotal determinant influencing the yield and characteristics of biobutanol production (Visioli et al., 2014; Huzir et al., 2018). Pursuing sustainable energy solutions necessitates a thorough exploration of diverse substrates, as comprehending their relative merits becomes crucial (Huzir et al., 2018). Therefore, it is crucial to conduct research that explores various substrates for biobutanol production using *Clostridium* species, emphasizing their roles in this process. This includes comparing and optimizing the biobutanol production results from each raw material, as summarized in Table 1.

**Table 1:** Comparison of feedstocks efficiency.

Microorganisms	Substrates	Pretreatment	Fermentation Conditions	Solvent Productions		References
				Butanol (g/L)	ABE (g/L)	
*C. saccharoperbutylacetonicum* N1–4	Switchgrass	Acetic acid	Solid-state fermentation (SSF)	8.6	13.9	Wang et al. (2019)
*C. acetobutylicum* ATCC 824	Corn starch	N/A	SSF	11.2	21.5	Wang et al. (2023)
*C. beijerinckii* P260	Barley straw hydrolysate	Mechanical + acidic pretreatment	Fermentation with product removal by gas stripping	30.8	47.20	Qureshi et al. (2014)
*C. acetobutylicum* L7	Corn stover	Acid	Batch	10.8	18.2	Wu et al. (2021)
*C. acetobutylicum* GX01	Sugarcane bagasse	Alkali	Batch	14.17	21.11	Pang et al. (2016)
*C. beijerinckii* P260	Food waste (mashed potatoes, sweet corn, and white bread)	N/A	Batch	± 9	14.2	Huang et al. (2015)
*C. beijerinckii* B-466	Agro-industrial waste (starch industry wastewater)	H_2_SO_4_	Batch	11.4	18.83	Maiti et al., (2016)
*C.beijerinckii* DSM-6422	Brown algal (L. *digitata*)	Enzymatic hydrolysis	Batch	7.16	N/A	Hou et al. (2017)
*C. tyrobutyricum* Ct-pMA12G	Brown algal (*S. japonica*)	Ultrasonic-assisted acid hydrolysis	Batch	12.15	N/A	Fu et al. (2021)
*C. beijerinckii*	Green algal (*U. lactuca*)	H_2_SO_4_ + hydrolases	N/A	3	4.5	van der Wal et al. (2013)
*Clostridium* sp. strain WK	Red algal (*G. amansii*)	H_2_SO_4_	Batch	3.46	N/A	Hong et al. (2019)
*C. saccharobutylicum* DSM 13864	Cane molasses	H_2_SO_4_	Batch (two-stage semi continuous fermentation)	8.79	13.15	Ni et al. (2012)
*C. acetobutylicum*	Municipal solid wastes (waste compost plant)	Ethanol organosolv	Batch	8.57	13	Farmanbordar et al. (2018b)
*C. acetobutylicum*	*Chlorella vulgaris* JSC-6	Sequential alkali pretreatment and acid hydrolysis	Batch	13.1	N/A	Wang et al., (2016)
*C. acetobutylicum* ATCC 824	*Chlorella sorokiniana*	Acid pretreatment followed by enzymatic hydrolysis	Batch	4.6	7.2	Yang et al. (2023)
*C. acetobutylicum*	*Borodinellopsis texensis* CCALA 892	Acid hydrolysis	N/A	3.63	N/A	Onay (2020)
*C. carboxidivorans* strain P7	Syngas (CO:H_2_:CO_2_ [70:20:10])	N/A	Batch	1.09	N/A	Phillips et al. (2015)
*C. aceticum* DSM 1496 and *C. kluyveri* DSM 555	Syngas	N/A	Fed-Batch	0.951	N/A	Fernandez-Blanco et al. (2022)

N/A: not available in the references.

### 3.1 Agricultural waste

Agricultural waste comprises materials produced throughout different phases of the farming process, encompassing final products, secondary products, and unused raw materials (Palaniswamy et al., 2023). Agricultural waste is categorized into crop residues, plant residues, industrial waste, animal manure, and food waste (Awogbemi and Kallon, 2022). Rich in lignocellulose, agricultural residues primarily contain cellulose, lignin, hemicellulose, and extractives (Saini et al., 2014; Awogbemi and Kallon, 2022). These resources are characterized as carbon-neutral, sustainable, and cost-effective materials (Guo et al., 2022).

The typical structural composition of lignocellulosic biomass generally consists of 30 %–50 % cellulose, 15 %–35 % hemicellulose, and 10 %–20 % lignin (Dharmaraja et al., 2020). Hexoses can be formed from cellulose, which is a non-branched homopolysaccharide made up of d-glucopyranosyl units, while hemicellulose is a branched heteropolysaccharide made up of hexose and pentose sugar residues (Escobar et al., 2020; Guo et al., 2022). Lignin, consisting of phenylpropane linked through various inter-unit connections, plays a crucial role in binding the lignocellulosic matrix and reinforcing the cell walls (Vinod et al., 2023; Guo et al., 2022). Consequently, the removal of lignin is deemed necessary for biobutanol production from lignocellulose before the hydrolysis of cellulose and hemicellulose (Guo et al., 2022).

The production of butanol by *Clostridium* utilizing lignocellulose involves four primary steps: pretreatment (where the complex structure of lignocellulosic biomass is broken down), hydrolysis (which provides fermentable monomers), fermentation, and distillation (Magalhaes et al., 2018; Guo et al., 2022). However, the unique compositions and structures of various biomass types require distinct behaviors and tailored processing techniques (Chen et al., 2024). Essentially, pretreatment processes are categorized into four general types: biological (e.g., white-rot fungi), physical or mechanical treatment (e.g., ultrasound, pyrolysis, microwave), chemical (e.g., organic solutions, base, dilute acid, ozone), and physiochemical (e.g., wet oxidation, hot water, steam explosion) (Magalhaes et al., 2018; Sun et al., 2019; Guo et al., 2022).

Optimal pretreatment parameters induce a morphological transformation in plant fibers, progressively enlarging pore size and markedly enhancing enzymatic hydrolysis efficiency, potentially tripling to tenfold enhancement before enzymatic hydrolysis initiation (Shangdiar et al., 2022). The effectiveness of sugar extraction from hydrolysis and its subsequent utilization in fermentation relies on pretreatment, which also produces inhibitory compounds (Sun et al., 2019). Detoxification of these compounds is essential to achieving increased product yields (Ebrahimian et al., 2023). However, approaches including simultaneous saccharification and fermentation (SSF), separate hydrolysis and fermentation or co-fermentation (SHF or SHCF), and consolidated bioprocesses (CBP) aimed at enhancing biofuel productivity are associated with high production costs and intensive energy requirements (Sun et al., 2019).

Moreover, innovative strategies for pretreating biomass play a pivotal role in achieving efficient bioenergy production (Wang et al., 2019; Quevedo-Amador et al., 2024). For most known pretreatment processes (particularly for physico-chemical and chemical processes), chemical reagents (including dilute acids, bases, ionic liquids, or organosolv) are typically employed as catalysts to break down the tough structure of biomass (Wang et al., 2019; Sagar et al., 2023). The effectiveness of these reagents varies among plant types and wastes (Raita et al., 2021). For example, research by Xu et al. (2020) utilized environmentally friendly ethylamine-based eutectic solvents and lactic acid in the pretreatment of lignocellulosic biomass from corn cobs. These reagents yielded a high sugar concentration of 53.5 g/L. Alkaline treatment increased the cellulose content to nearly 34 % and reduced the lignin content by 15 %, making corn cobs more suitable for ABE fermentation.

Another study by Jannah et al. (2019) demonstrated that the use of 7 % ammonia resulted in the highest lignocellulosic content, with 69.68 % cellulose, 14.39 % hemicellulose, and 9.15 % lignin. However, this pretreatment process produces by-products (inhibitors) that can be categorized into three main groups: phenolic compounds (p-hydroxybenzoic acid, ferulic acid, vanillic acid, p-hydroxybenzaldehyde, vanillin, etc.), furan derivatives (5-hydroxymethylfurfural, 2-furoic acid, furfural, etc.), and weak organic acids (formic acid, levulinic acid, acetic acid, etc.) (Luo et al., 2020). These inhibitors adversely affect microbial growth, increase lag phase length, cause cell density loss (Kumar et al., 2020), substrate utilization, and fermentation (Ojo, 2023). Although strategies exist to remove these inhibitors, they also lead to the loss of fermentable sugars, which is economically impractical (Luo et al., 2020).

Furthermore, Wang et al. (2019) utilized acetic acid in switchgrass pretreatment, with the resulting reagent being usable for biobutanol production, reducing costs, and avoiding fermentation inhibition. Pretreatment with acetic acid produces phenolic inhibitors at lower levels than strong chemical reagents but detoxification is still required before fermentation. Detoxification methods using activated carbon can remove 5-hydroxymethylfurfural (HMF) and furfural compounds by 50–60 % and total phenolic compounds by 50 %.

Moreover, various constraints hinder biobutanol production by *Clostridium* from lignocellulosic biomass hydrolysates, such as the strains' incapacity to tolerate inhibitors found in the hydrolysates, issues related to xylose consumption, strain intolerance to elevated butanol concentrations, and low titers (Magalhaes et al., 2018; Palaniswamy et al., 2023). Microorganism engineering needs to be conducted to enhance resistance to inhibitor compounds (Kumar et al., 2020). Amidst advancements in the biofuel field, Magalhaes et al. (2018) addressed issues such as inhibitors and optimized the utilization of xylose from sugarcane bagasse hydrolysate. It is noted that *C. saccharobutylicum* DSM 13,864 excels at fermenting lignocellulosic hydrolysate, generating substantial quantities of acetone, n-butanol, and ethanol.

Additionally, Qureshi et al. (2014) improved ABE fermentation by incorporating concentrated sugar solutions from barley straw and corn stover hydrolysates, employing the *C. beijerinckii* P260 strain. Remarkably, the culture efficiently utilized 99.4–100 % of sugars in these hydrolysates, limited only by potential toxicity at concentrations surpassing 100 g/L. The success can be attributed to the synergistic utilization of concentrated hydrolysates and simultaneous product recovery, outperforming controls based on glucose fermentation. The integrated system not only improved sugar utilization and ABE productivity, but it also demonstrated solvents' superior specific productivities.

It was reported that lignocellulosic biomass hydrolysates often lack essential components needed by microorganisms, necessitating the addition of nutrients that can increase raw material costs (Chen et al., 2024). Additionally, findings by Huang et al. (2015) reveal that leveraging *C. beijerinckii* P260 on food waste (such as mashed potatoes, sweet corn, and white bread) effectively slashes raw material costs by enhancing productivity and minimizing residual sugars. Fermenting food waste at elevated concentrations and using vacuum peeling technology possess the potential to curtail energy consumption, water usage, as well as process volume and equipment, consequently leading to substantial reductions in butanol production costs.

Jang and Choi (2018) conducted a techno-economic investigation into bio-butanol production, incorporating technical data from laboratory operations, pilot plant trials, and demonstration plant designs. The process entails concentrated acid pretreatment and hydrolysis to produce sugar from lignocellulosic biomass, along with continuous fermentation systems for butanol production from sugar using *Clostridium acetobutylicum* ATCC 824 capable of co-fermentation of C6 and C5. At a plant capacity of 80,000 tons per year, the total production cost for biobutanol, including by-product credit, is US$1427/ton, with a corresponding minimum selling price of US$1693/ton. It is worth mentioning that the production costs need to be reduced to US$770/ton (54 %) to ensure commercial viability by aligning the minimum selling price with the average market price of petroleum-based butanol at US$1207.8/ton.

Research on increasing biobutanol production is continuously evolving, particularly in terms of commercialization, research and development, and pilot-scale studies. According to Strömberg et al. (2014), agricultural waste has been the foundation for the biorefinery concept since the establishment of the Dokshukino plant in the Soviet Union in 1962. Although several ABE fermentation plants were closed by the late 1980s, trials conducted in China in 2007 demonstrated a potential sugar conversion of up to 80 % for ABE production from straw through steam hydrolysis. Laihe Chemical Ltd. Co. in Songyuan City, Jilin Province, commenced butanol production from straw in 2010 with an annual capacity of 300,000 tons, eventually joining forces with Laihe Rockley Bio-Chemicals Co., Ltd. to construct two additional ABE fermentation plants. Innovative steps, such as those taken by Cobalt Technologies and Green Biologics, involved genetically modified clostridial strains for n-butanol production. However, despite successful trials, Cobalt Technologies ceased operations in 2015, and Green Biologics also had to close its plant in Little Falls in 2019 due to funding constraints (Gehrmann and Tenhumberg, 2020).

### 3.2 Municipal solid waste

Municipal solid waste (MSW) comprises a diverse mixture of non-biomass materials, encompassing easily combustible substances, plant and animal products, paper, oil, and other waste materials rich in lignocellulose (Manirethan et al., 2022; Palaniswamy et al., 2023). MSW offers a cost-effective resource with potential for valorization into higher-value products, serving as feedstocks for both sugar production and fermentation into various biofuel products (Farmanbordar et al., 2018b; Thompson et al., 2019). The utilization of MSW as a substitute for gasoline has the potential to significantly reduce greenhouse gas emissions by 29.2 % to 86.1 % (Palaniswamy et al., 2023).

However, the complex composition of the organic fraction of municipal solid waste (OFMSW) differs from agricultural and forestry by-products, which typically demonstrate consistent composition profiles and generally lack contaminants such as toxic metals (Dornau et al., 2020). It has been demonstrated that the presence of phenolic compounds, including tannins, in high concentrations hinders butanol production (Mirfakhar et al., 2017; Farmanbordar et al., 2018a). The extraction of these phenolic compounds with ethanol is commonly conducted prior to the butanol production process (Farmanbordar et al., 2018a). In addition to their toxic properties, the combination of lignocellulose and starch introduces additional obstacles in the production of butanol from the OFMSW due to their differing chemical properties (Farmanbordar et al., 2018b). The utilization of MSW to produce biobutanol involves several stages, including pretreatment, hydrolysis, fermentation, and distillation (Meng et al., 2019; Ashani et al., 2020).

Farmanbordar et al. (2018b) reported that ethanol organosolv pretreatment resulted in a substantial increase in total solvent production, particularly butanol, derived from waste compost plants. This pretreatment can remove lignin by 28.7–84.5 % and dissolve starch with recovery rates ranging from 29 % to 94 %. Furthermore, this method is effective in eliminating most phenolic compounds and tannins, with reductions of 74–96 % and 91–98 %, respectively. Another study by Farmanbordar et al. (2020) employed dilute acid in the initial treatment and *C. acetobutylicum* NRRL B-591 for the fermentation process, involving urban waste collection plants, wastepaper, and garden waste. The composition of lignocellulosic and starch materials in the substrate undergoes significant alteration due to dilute acid treatment, resulting in the removal of hemicellulose and xylan content from garden waste and rice straw, with removal percentages ranging from 75 % to 94 %. It has been observed that this process yields higher ABE yields compared to hydrolysates from individual substrates. Pre-treatment with diluted acid offers cost-effectiveness, lower aggressiveness, and maximum sugar recovery from hemicellulose (Phwan et al., 2019; Sagar et al., 2023).

However, the use of inorganic acid can lead to equipment corrosion and environmental issues associated with acidic waste streams (Abolore et al., 2024). Meng et al. (2019) developed the latest pretreatment technology, autoclaving, to replace conventional dilute acid for processing unsorted waste. Autoclaving with sorting, anaerobic digestion, and composting has the least environmental impact in terms of eutrophication and global warming potential. The evaluation of ethanol, acetone, butanol, and hydrogen production reveals yield of 1.5 kg ethanol, 5.7 kg acetone, 12.2 kg butanol, and 0.9 kg hydrogen per ton of MSW, resulting in a net greenhouse gas reduction of 115 % compared to gasoline.

Moreover, research by Thompson et al. (2019) demonstrated that MSW paper and MSW grass clippings mixed with corn stover can be utilized to produce biofuel at a production cost ranging from US$72.83 to US$76.26. Both materials meet the National Renewable Energy Laboratory (NREL) set production cost target of less than US$85.51/ton, calculated on a 2016-year basis. In summary, the utilization of municipal solid waste presents a viable pathway for sustainable biofuel production, although challenges such as complex composition and phenolic compounds require innovative solutions for efficient conversion. Meanwhile, Ashani et al. (2020) conducted another study on the technical and economic analysis of a new process for ABE production from MSW, reveals that gas stripping and pervaporation enhance ABE production, reducing distillation costs, and offer promising payback periods. This confirms that utilizing of MSW with gas stripping and pervaporation approaches has significant potential to be an economical solution in ABE production. Nevertheless, challenges such as complex composition and phenolic compounds remain the focus for seeking innovative solutions to enhance conversion efficiency.

### 3.3 Macroalgae

Macroalgae, colloquially known as seaweed, are multicellular algae possessing chlorophyll but devoid of true stems and roots (Milledge et al., 2014; Moreira et al., 2021). These macroalgae possess the capacity for rapid growth, are a replenishable resource, absorb CO_2_ from the surroundings, and take in inorganic elements or surplus nutrients from seawater (Chen et al., 2015; Schultze-Jena et al., 2022). The growth rate and amount of biomass per unit surface area attainable in seaweed farms exceed those of terrestrial plants, primarily due to the minimal energy requirements for production (Moenaert et al., 2023).

Furthermore, cultivating macroalgae does not necessitate agricultural land and numerous species thrive in brackish or saline water, thereby avoiding competition for the land and freshwater necessary for food production (Milledge et al., 2014; Milledge and Harvey, 2016; Simon et al., 2022). The biomass of macroalgae comprises a range of sugar polymers with low or no lignin content, necessitating less severe pretreatment (Hou et al., 2017; Sharmila et al., 2021). The residues of biomass post-conversion can be employed for heating, fertilizers, and diverse fuel production purposes (Sharmila et al., 2021).

In comparison to microalgae, macroalgae offer various advantages, including ease of harvest and a less complex algae separation system in aquaculture environments (Liu et al., 2023). Macroalgae show potential for bio-based products and fuels as part of the shift towards a blue bioeconomy, but they encounter obstacles in becoming commercially viable (Milledge and Harvey, 2016). These hurdles include challenges in cultivating and harvesting macroalgae, establishing consistent supply chains, species selection issues, and the complex nature of hydrolysis and conversion procedures (Sharmila et al., 2021). Scaling up biorefinery techniques for macroalgae faces difficulties in effectively integrating various processing components (Sharmila et al., 2021; Suhartini et al., 2024). Moreover, the increased use of freshwater during bioprocessing stages raises concerns amid global freshwater scarcity, although incorporating seawater into certain processes holds promise (Sharmila et al., 2021; Musie and Gova, 2023). Despite ongoing research at the laboratory level, the practical application of these technologies on a larger scale remains uncertain, underscoring the importance of further refining and validating holistic macroalgal biorefinery methods (Sharmila et al., 2021).

According to their pigmentation, macroalgae fall into three primary groups: green (Chlorophyceae), red (Rhodophyceae), and brown (Phaeophyceae) (Erniati et al., 2023). Brown macroalgae predominantly flourish in waters with very cold or extremely cold temperatures, whereas red algae mainly grow in the inter-tropical zone. Green algae can be found in various aquatic environments (Chen et al., 2015). Macroalgae possess abundant water-soluble carbohydrates (25 %–50 %), proteins (7 %–15 %), and lipids (1 %–5 %) (Sharmila et al., 2021). They display complex structures, with brown macroalgae containing a substantial amount of alginate and sulfated polysaccharides, red macroalgae containing agar and carrageenan, and green macroalgae exclusively having protein-bound polysaccharides (Dziergowska et al., 2022). Consequently, the production of biobutanol from macroalgae involves various processes, including pretreatment, hydrolysis, fermentation, and separation (Schultze-Jena et al., 2022; Llano et al., 2023). Ideal pretreatment methods need to be simple, enable effective inhibitor removal, possess a large reactive surface area, and reduce cellulose crystallinity, while energy requirements and costs are considered (Salaeh et al., 2019). The cost reliability of butanol derived from the macroalgae *Ulva rigida*, fermented by *C. acetobutylicum,* has been demonstrated, with a production rate of 2,667 kg/hour of butanol (purity 99.8 %) (Llano et al., 2023). Therefore, macroalgae are recognized as one of the most promising sources of biofuel for the future (Menetrez, 2012).

Various categories of macroalgae have been extensively investigated for their potential in butanol production, including *U. lactuca* (van der Wal et al., 2013; Bikker et al., 2016), *Saccharina latissima* (Schultze-Jena et al., 2022), *S. japonica* (Fu et al., 2021), *Rhizoclonium* spp. (Salaeh et al., 2019), and *Gelidium amansii* (Hong et al., 2019). There has been increased focus on brown macroalgae for the advancement of sustainable biofuels due to their notably higher efficiency compared to cyanobacteria or red algae (Song et al., 2015). Brown macroalgae possess a relatively substantial capacity for converting photons, facilitating quicker biomass synthesis (Mahmood et al., 2022). Moreover, they comprise components that can undergo fermentation at relatively elevated concentrations (Schultze-Jena et al., 2022).

Research carried out by Hou et al. (2017) using hydrolysate from seaweed demonstrated that *C. beijerinckii* DSM-6422 could utilize glucose, mannitol, and even glucan from *Laminaria digitata* as substrates. Fermentation of the hydrolysate yielded higher butanol compared to the control, achieving a butanol:ABE molar ratio of 0.85. Furthermore, Fu et al. (2021) observed that acid-assisted ultrasonic pretreatment of *S. japonica* hydrolysate could enhance the processing, resulting in the highest butanol yield (0.26 g/g) and productivity (0.19 g/L⋅h). The genetically engineered strain *C. tyrobutiricum* Ct-pMA12G demonstrated increased butanol tolerance and enhanced butanol production, reaching the highest levels ever reported for macroalgal biomass. Salaeh et al. (2019) investigated the use of sulfuric acid (H_2_SO_4_) as an initial treatment for *Rhizoclonium* spp. residue substrates, resulting in the highest sugar release of 558 mg/g, with fermentation by *C. beijerenckii* TISTR 1461 yielding 135 mg of butanol per gram of sugar. The sulfuric acid pretreatment proved to enhance cellulose and hemicellulose hydrolysis efficiency by increasing the surface area of seaweed waste and improving the final glucose yield for fermentation.

### 3.4 Microalgae

Microalgae emerge as a promising alternative reservoir for butanol generation owing to their ability to accumulate substantial quantities of fermentable carbohydrates, sequester CO_2_, demonstrate accelerated growth rates relative to terrestrial plants, and metabolize both inorganic and organic carbon into biomass (Shanmugam et al., 2021; Khan et al., 2023; Yang et al., 2023). Microalgal biomass typically consists of 20–50 % lipids, 20–40 % carbohydrates, and 60 % protein (Shanmugam et al., 2021). Strains of marine or brackish water microalgae have a minimal freshwater footprint and can be cultivated in non-soil areas under extreme climatic conditions (Mahata et al., 2022; Khan et al., 2023). Microalgae cultivation can also utilize wastewater sources from palm oil industries (Low et al., 2021) or household sewage systems (Ali et al., 2022; Osman et al., 2023; Ghaffar et al., 2023). Microalgal species from various families exhibit similar properties and characteristics, enabling beneficial commercial applications (Sundaram et al., 2023).

Furthermore, specific strains of microalgae, such as *Chlorella vulgaris, Coelatrella* spp., *Scenedesmus obliquus, Scenedesmus dimorphus, Chlamydomonas reinhardtii, Dunaliella* spp., and *Spirulina* spp., exhibit the capability to accumulate significant quantities of carbohydrates, lipids, and proteins, thereby making them excellent candidates as raw materials for biofuel production (Khan et al., 2023). In addition, Sohedein et al. (2020) highlighted the significance of thraustochytrids, a type of oleaginous microorganism often referred to as microalgae due to their rich biomass, lipids, and carotenoids. These organisms have attracted considerable interest for their potential in biodiesel production, biomedical applications, and nutraceuticals. Their capacity to produce high levels of polyunsaturated fatty acids (PUFAs) and saturated fatty acids (SFAs) positions them as a promising renewable biofuel source.

Despite these advantages, the production of butanol from microalgae still faces challenges, primarily due to the relatively high production costs (Wang et al., 2017; Ganesan et al., 2020). Wang et al. (2017) reported that the production cost of microalgae remains significant, reaching US$434/1000 kg of biomass or US$108/100 kg of microalgal sugar. Additionally, research on microalgae for butanol production encounters difficulties in obtaining glucose due to the rigid cell walls, restricting access to intracellular starch (de Souza et al., 2020). Consequently, pre-treatment processes become essential for releasing starch for subsequent hydrolysis (Yang et al., 2023). Following this, either a chemical process involving mineral acid and alkaline solutions or an enzymatic saccharification process with enzymes is employed to hydrolyze the released starch into fermentable glucose (Chen et al., 2013). The efficacy of using microalgal biomass as a biofuel raw material depends on the efficiency of the processing for fermentable glucose production (Yang et al., 2023).

Wang et al. (2016) reported that a sequential alkali pretreatment and acid hydrolysis method can enhance butanol production from *C. vulgaris* by effectively eliminating potential fermentation inhibitors, particularly protein-related substances found in microalgal hydrolysates. In addition, biomass processing encounters significant challenges, particularly regarding the high costs associated with drying processes, as shown in a study conducted by Yang et al. (2023). This experiment involved fresh biomass, demonstrating that it can be directly utilized for acid and enzymatic hydrolysis processes. The results revealed an achievement of 88.5 % for acid hydrolysis and 75 % for enzymatic hydrolysis, comparable to results obtained from freeze-dried biomass. Furthermore, the glucose yield was higher compared to the use of oven-dried biomass. Hydrolysates from pretreatment with dilute sulfuric acid and enzymatic hydrolysis were fermentable by *C. acetobutylicum* ATCC 834, producing butanol and ABE yields comparable to liquid starch.

Another study conducted by Huang et al. (2015) examined the ability of *Borodinellopsis texensis* CCALA 892 to produce biobutanol in synthetic wastewater. Microalgae samples cultivated in 25 % wastewater showed the highest biomass productivity, carbohydrate concentration, protein concentration, and bio-butanol content. In conclusion, microalgae show promise for butanol production, yet addressing cost and extraction challenges calls for innovative pre-treatment and hydrolysis methods (Wang et al., 2017; Ganesan et al., 2020).

The study conducted by Villacreses-Freire et al. (2022) on life cycle assessment (LCA) for biobutanol production using genetically modified *Synechocytis* PCC6803 demonstrates increased productivity and greater environmental impact. Nevertheless, the electricity requirements for cultivation and harvesting outweigh the advantages. Considering scenarios, the use of renewable electricity can result in a better climate gas balance, reducing greenhouse gas emissions to 3.1 kg CO_2_ eq/kg biobutanol, approximately 20 % higher than fossil reference gas emissions (2.45 kg CO_2_ eq/kg 1-butanol). Additional research and development efforts are necessary to surmount these barriers and maximize the contribution of microalgae to sustainable biobutanol production.

### 3.5 C1 gases (syngas)

Butanol can be produced through raw materials that are gasified into syngas, a mixture of hydrogen (H_2_), carbon dioxide (CO_2_), and carbon monoxide (CO) (He et al., 2022c). The gas mixture can be obtained from biomass gasification or waste, including industrial gas waste such as steel production process waste (Fernandez-Naveira et al., 2017a). Syngas offers dual benefits: fermentation for carbon capture (mitigating pollution) alongside energy production (Lanzillo et al., 2020). Only a few microorganisms, primarily clostridia, have been identified for their ability to utilize syngas as a substrate to generate medium-chain fatty acids or alcohols, such as butyric acid, butanol, hexanoic acid, and hexanol (Zhang et al., 2016; Lanzillo et al., 2020).

*Clostridium* spp. are the primary functional species due to their specific enzyme, carbon monoxide dehydrogenase (CODH), which is used to overcome carbon monoxide toxicity by converting it into CO_2_, subsequently reduced to acetyl-CoA with the assistance of the enzyme acetyl coenzyme A (acetyl-CoA) synthase (ACS) (He et al., 2022b). *C. carboxidivorans, C. ljungdahlii, C. ragsdalei,* and *C. autoethanogenum* can convert CO, CO_2_, and H_2_ into acids, ethanol, and butanol through the Wood-Ljungdahl pathway (WLP) metabolism (Rückel et al., 2021). *Clostridium carboxidivorans* has a complete gene cluster related to the WLP, excluding the acetone pathway, and is capable of producing butanol and hexanol, which have not been found in other bacterial species (Fernandez-Naveira et al., 2017a). Only a few *Clostridium* strains, such as the pure culture of *C. carboxidivorans* and the co-culture of *C. autoethanogenum* and *C. kluyveri,* can produce these alcohols from 100 % CO (He et al., 2022c). In contrast to biochemical methods, syngas fermentation presents benefits by transforming all biomass constituents, including lignin, into syngas, thus amplifying product yield from equivalent biomass quantities (Sun et al., 2019). Although H_2_, CO, and CO_2_ are the primary components in numerous industrial waste gases and syngas, the presence of additional compounds such as acetylene, NO, sulfur, and ammonia can inhibit enzyme activity and bacterial growth in the fermentation process (Fernandez-Naveira et al., 2017a).

The biobutanol production process using syngas involves several stages. Firstly, biomass gasification into syngas (a mixture of H_2_, CO, and CO_2_), followed by syngas fermentation by acetogenic microorganisms such as *Clostridium,* and distillation (Daniell et al., 2012; Fernandez-Naveira et al., 2017a; He et al., 2022b). Syngas has the potential to be transformed into hydrocarbon liquid fuels using the Fischer-Tropsch process and into alcohols, organic acids, and various other chemicals through syngas fermentation. The Fischer-Tropsch process utilizes metal catalysts at high temperatures and pressures, requiring a high H_2_:CO molar ratio, typically ranging from 2:1 to 3:1 (Sun et al., 2019). The fermentation process of butanol with syngas faces challenges, such as the low solubility of CO and other compounds in water, which reduces substrate transport to the liquid phase in the bioreactor and affects production yield (Fernandez-Naveira et al., 2017a; Lanzillo et al., 2020; Elisiário et al., 2021; He et al., 2022b). The use of membrane systems and microbubble spargers can be employed to enhance more efficient mass transfer (Lanzillo et al., 2020; Elisiário et al., 2021). Pressurized bioreactors and packed-bed bioreactors, such as biofilters or biotrickling filters, can improve gas solubility, mass transfer, and microbial substrate utilization, although this will also increase operational costs (Fernandez-Naveira et al., 2017a).

Additionally, solvent toxicity becomes a significant factor in butanol fermentation, as bacterial cells rarely tolerate high concentrations of butanol (Fernandez-Naveira et al., 2017a; Lanzillo et al., 2020). CO plays a crucial role in the availability of reducing equivalents and carbon conversion (Lanzillo et al., 2020). Increasing the partial pressure of CO results in higher cell concentrations, increased growth-associated ethanol production, and decreased acetate formation (Dürre, 2016). Research by Lanzillo et al. (2020) indicates that CO concentration affects the maximum cell growth of *C. carboxidiovorans* and CO conversion, with the best results obtained at an initial CO pressure of around 1.7 atm. This is essential for designing and controlling large-scale fermenters in biobutanol production using syngas. Temperature control is crucial in alcohol production to avoid "acid crashes", resulting in low alcohol production. At 25 °C, higher concentrations of ethanol, butanol, and hexanol were found compared to 37 °C. Another approach with limited media and an incubation temperature of 37 °C resulted in higher alcohol concentrations. Similar butanol concentrations were also found in continuous-operation bioreactors at controlled temperatures and pHs (Dürre, 2016).

In addition, the disadvantages of syngas fermentation encompass restrictions in gas-liquid mass transfer, diminished productivity, elevated production expenses, and the generation of impurity gases that may influence microbial metabolism (Sun et al., 2019; Rückel et al., 2021). Nevertheless, research into syngas fermentation continues to advance. For example, Phillips et al. (2015) found that careful adjustment of nutrients to enhance the production of higher alcohols could reduce production expenses. When grown in a CO:H_2_:CO_2_ (70:20:10) environment, *C. carboxidivorans* can produce the best-quality butanol. Essential elements for this process include ensuring low CO pressure and regulating mass transfer to improve alcohol yield. Fernández-Blanco et al. (2022) investigated the integration of syngas fermentation and chain elongation methodologies to produce medium-chain fatty acids (MCFAs) and bioalcohols, aligning with the circular economy concept. They utilized a novel co-culture of *C. aceticum* and *C. kluyveri* in stirred tank bioreactors, efficiently metabolizing syngas at nearly neutral pH levels, resulting in significant yields of n-butyrate, n-caproate, and n-butanol. Additionally, *C. aceticum* demonstrated its capability to produce n-butanol from CO within this integrated framework.

Piccolo and Bezzo (2009) conducted a comparative techno-economic analysis (TEA) between the enzymatic hydrolysis fermentation (EHF) and the gasification-syngas fermentation (GF) processes, which have a capacity of 2030 metric tons per day of raw material and a production cost of US$80.13 per metric tons. They calculated the minimum ethanol selling price (MESP) for GF and EHF to be US$1.07 and US$1.01 per liter, respectively. From the comparison results, it can be observed that the gasification-syngas fermentation process has a slightly higher MESP compared to the enzymatic hydrolysis fermentation process. Several companies, including LanzaTech, Coskata, and INEOS Bio, have pursued the scale-up and commercialization of syngas fermentation processes, effectively utilizing autotrophic acetogens to convert syngas into ethanol (Dürre, 2016; Fernandez-Naveira et al., 2017a; Sun et al., 2019). For instance, INEOS Bio has established a plant capable of generating 8 million gallons of ethanol each year, while LanzaTech has developed demonstration plants in China and New Zealand with capacities of up to 100,000 gallons each year, using waste gases from steel mills as feedstock. The recent partnership between LanzaTech, ArcelorMittal, and Primetals Technologies marks a significant step in constructing commercial plants with capacities of up to 47,000 tons per year. Apart from waste gases from steel mills, other industrial waste gases and solid waste gasification can also be used as syngas sources, providing potential for producing high-value chemicals such as butanol (Dürre, 2016; Fernandez-Naveira et al., 2017a).

Patents related to the development of the integration processes of gasification, fermentation, and product recovery ensure efficient operation and increased productivity. The design of bioreactors with enhanced gas-liquid mass transfer is crucial for the commercialization of syngas fermentation. The use of automatic control in the syngas fermentation process has been patented by LanzaTech and Oklahoma State University, enhancing the productivity of final products without an excessive supply of substrates. This method also reduces operational costs and accelerates the start-up process to achieve sustainable production in a shorter period of time (Sun et al., 2019).

**Fig. 1:**
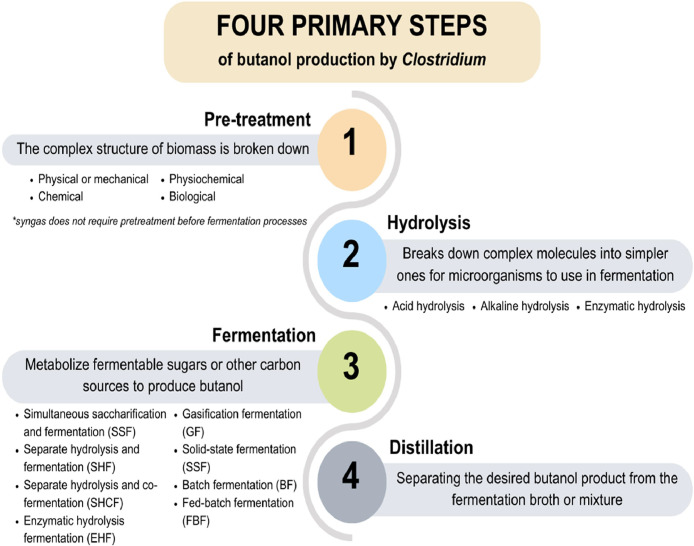
The summary of biobutanol production process from underutilized substrates.

There are various raw material options for producing biobutanol using *Clostridium*, ranging from agricultural waste and municipal solid waste to macroalgae, microalgae, and syngas. Each raw material has its own unique characteristics, offering different potential contributions to biobutanol production. Table 2 presents a direct comparative summary of the efficiency of each substrate. Fig. 1 summarizes the biobutanol production process from underutilized substrates.

**Table 2:** Summary of the comparative efficiency of fermentation using various raw materials.

Substrates	Compositions	Pre-treatments	Advantages	Challenges	Cost
Agricultural wastes	Rich in lignocellulose, it contains cellulose, hemicellulose, and lignin	Physical, chemical, physiochemical, and biological processes	Carbon-neutral, sustainable, does not compete with food, and is a cost-effective resource for biobutanol production	Xylose consumption issues	Total production cost of US$1427/ton
Municipal Solid Wastes (MSW)	A diverse mixture of materials, including easily combustible substances, plant and animal products, paper, oil, and lignocellulosic waste	Physical, chemical, physiochemical, and biological processes	Potential greenhouse gas emissions reduction and the production of biofuels from waste materials	Complex composition, the presence of phenolic compounds that hinder butanol production, and the differing chemical properties of lignocellulose and starch	MSW paper and MSW grass clippings mixed with corn stover can be utilized to produce biofuel at a production cost ranging from US$72.83 to US$76.26
Macroalgae	Abundant water-soluble carbohydrates, proteins, and lipids, with complex structures containing specific polysaccharides depending on the type of algae	Physical, chemical, physiochemical, and biological processes	Rapid growth rates, minimal energy requirements for production, absence of competition for agricultural land or freshwater, ease of harvest, and algae separation in aquaculture environments	Cultivation, distribution logistics, species selectivity, and scalability issues	–
Microalgae	Rich in fermentable carbohydrates, lipids, and proteins	Physical, chemical, physiochemical, and biological processes	Ability to accumulate fermentable carbohydrates, sequester CO_2_, demonstrate accelerated growth rates, and utilize both inorganic and organic carbon into biomass. They can be cultivated in non-soil areas under extreme climatic conditions and can utilize wastewater sources	High production costs and obtaining fermentable glucose due to their rigid cell walls	Total production cost US$434/1000 kg of biomass or US$108/100 kg of microalgal sugar
Syngas	CO, CO_2_, and H_2_, enable microbial conversion into medium-chain fatty acids or alcohols	Does not require pretreatment before fermentation processes	The utilization of waste gases as feedstock, potential for high-value chemical production, and ongoing advancements in commercialization efforts by companies such as INEOS Bio and LanzaTech	Restrictions in gas-liquid mass transfer, diminished productivity, elevated production expenses, and the generation of impurity gases influence microbial metabolism	Total production cost $80.13 per metric ton; estimated MESP for GF and EHF at $1.07 and $1.01 per liter, respectively

- : No data.

## 4 Generation of feedstocks

First-generation biofuel sources are derived from consumable resources such as starch (potatoes, wheat, corn, and barley), sugar (sugarcane and beets), vegetable oil, and fats (Alalwan et al., 2019; Rai et al., 2022; Cavelius et al., 2023). These substrates can yield common biofuels like biodiesel, ethanol, biofuel gasoline, sugar alcohol, corn ethanol, and biogas (Rai et al., 2022). Despite their prevalence, these substrates face limitations, such as reduced production or yield (Rathour et al., 2018). Moreover, utilizing food crops for fuel raises significant concerns as it impacts food prices, food security, and the requirement of fertile land contributing to deforestation in tropical rainforests for biofuel cultivation, which releases more CO_2_ than the emissions saved by the biofuel (Moodley, 2021; Cavelius et al., 2023).

Second-generation biofuels are seen as a promising solution to the constraints presented by first-generation fuels, providing an opportunity for the development of an affordable, sustainable, and environmentally friendly fuel source (Rathour et al., 2018). These biofuel sources, derived from non-food crops and residual farm materials, are considered more sustainable as they do not compete with food resources, although effectively breaking down their complex structure poses a challenge (Alalwan et al., 2019; Cavelius et al., 2023). Biobutanol production from lignocellulosic biomass and other waste streams typically relies on clostridial fermentation, which is considered one of the most established and effective processes for butanol production (Cavelius et al., 2023). It is estimated that second-generation biofuels are projected to potentially fulfill about 30 % of the world's transportation energy needs (Malode et al., 2021). However, second-generation biofuel production is associated with higher costs, approximately US$1.65 per gallon, making it two to three times more expensive than conventional fuels (Aron et al., 2020). Additionally, these materials are scarce, requiring the integration of complementary technologies (Cavelius et al., 2023).

Third-generation biofuels, originating from photosynthetic microorganisms such as microalgae, are created through the utilization of carbon dioxide, light, and nutrients to generate biomass for biofuel synthesis (Behera et al., 2015; Sharmila et al., 2021). These biofuels present a more efficient energy option compared to their predecessors, characterized by shorter life cycles, lower land requirements, and faster growth and photosynthesis rates compared to terrestrial plants, and can be cultivated in seawater and wastewater, thus not competing with conventional agriculture (Wang et al., 2017; Rai et al., 2022). Macroalgae, reported as biomass, are capable of producing ethanol twice as high as sugarcane and five times higher than corn due to their low energy consumption (Maliha and Abu-Hijleh, 2022). However, third-generation sources, like microalgae, while holding promise, encounter obstacles due to their diminutive size and susceptibility to pH fluctuations (Alalwan et al., 2019; Cavelius et al., 2023). Commercializing biobutanol production from microalgae also incurs high capital and operational costs. Reported biomass production costs for microalgae are US$432/1000 kg of biomass or US$108/100 kg of microalgae sugar (Wang et al., 2017). Additionally, they require the highest energy inputs for production among biofuel alternatives (Pulyaeva et al., 2020).

Fourth-generation biofuel sources employ synthetic biology techniques to improve characteristics in organisms aimed at biofuel production (Alalwan et al., 2019; Cavelius et al., 2023). Within this framework, genetically engineered photosynthetic microorganisms, including cyanobacteria, algae, and fungi, serve as raw materials (Rai et al., 2022). Challenges in the advancement of fourth-generation biofuels include constraints in local genetic engineering infrastructure, potential product toxicity (Cavelius et al., 2023), health and environmental risks, concerns regarding legitimacy, and insufficient biomass production (Shokravi et al., 2021). Alternative methodologies such as random mutagenesis and electrobiofuel production also pose obstacles, requiring further refinement (Cavelius et al., 2023).

**Fig. 2:**
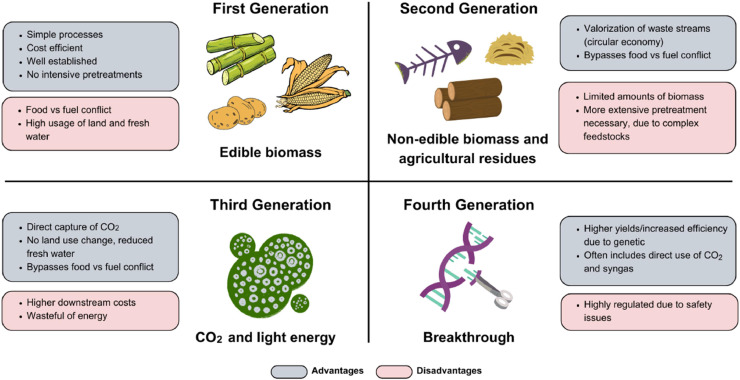
Feedstocks classification (Redrawn from Cavelius et al. (2023), with modification; the journal does not require permissions to use the materials).

The efficiency and environmental friendliness of raw material selection are crucial factors for successful biobutanol production (Visioli et al., 2014; Moon et al., 2016). Selecting raw materials is pivotal in influencing the overall cost-effectiveness and ecological impact of the biobutanol production process (Kumar and Gayen, 2011). Various raw material options, spanning first to fourth generations, can be utilized for biobutanol production, as illustrated in Fig. 2. Therefore, thoughtfully considering and choosing raw materials are essential steps to enhance the efficiency and environmental sustainability of the biobutanol production process.

## 5 Biotechnological advancements and limitations

Biobutanol has attracted attention due to its comparative advantages over other biofuels (Zheng et al., 2015). The prevalent substrate in ABE fermentation is lignocellulose sourced from agricultural waste (Moon et al., 2016; Carmona-Garcia et al., 2021). Nevertheless, the primary challenge associated with lignocellulosic substrates lies in their limited fermentable sugar release for microorganisms (Rana and Parul, 2018). Moreover, the economic feasibility of producing biofuel using plants as raw materials is presently inefficient in terms of cost, necessitating a much more effective approach to enhance biofuel production and make it economically viable and sustainable (Rai et al., 2022).

Biotechnological methods have improved butanol production efficiency by optimizing strain performance (Riaz et al., 2022). Strain development for butanol production has focused on aspects like butanol tolerance, improved substrate utilization, and increased butanol selectivity and productivity in lignocellulosic biomass waste (Palaniswamy et al., 2023). *Clostridium* strains typically yield less than 13 g/L of butanol (Liu et al., 2012; Lin et al., 2023) and tolerate butanol concentrations below 2 % (v/v) in batch fermentation (Liu et al., 2012; Xue et al., 2017). The toxicity of butanol to microbial systems is also complex and affects various cellular processes (Wang et al., 2020; Petrov et al., 2021). Butanol produced by *Clostridium* spp. inhibits nutrient transport into cells, disrupts membrane stability by fluidizing the phospholipid layer, and inhibits ATPase function, allowing ions to penetrate the membrane, thereby reducing cellular proton motive force and electrochemical potential (Peabody and Kao, 2016; Petrov et al., 2021).

Various genetic approaches, such as chemical mutagenesis and genome library construction, can enhance microbial resistance to butanol (Petrov et al., 2021). Transcriptomics is also useful for studying the molecular mechanisms of the butanol stress response. For instance, the overexpression of genes like *groESL* has been shown to significantly increase butanol tolerance and production in *C. acetobutylicum* (Du et al., 2022). Other research has identified cellular adaptive mechanisms, such as adjusting the ratio of saturated and unsaturated fatty acids, to reduce the toxicity of high concentrations of 1-butanol in cells (Peabody and Kao, 2016).

Naturally resistant species to butanol can serve as valuable genetic repositories to enhance the limited tolerance of natural butanol producers, or they can act as alternative hosts for clostridial ABE pathways and their synthetic equivalents (Petrov et al., 2021). Some bacteria exhibiting high tolerance to butanol include *Lactiplantibacillus plantarum*, with a tolerance range of 3 – 4 % (v/v) (Petrov et al., 2021), DT rob strain mutant of rob gene *Escherichia* coli with a tolerance of 1.25 % (v/v) (Wang et al., 2020), *Synechococcus elongatus* and *Saccharomyces ce*Re*visiae* with a tolerance range of 0.5 – 2 % (Liu et al., 2021a), *Bacillus subtilis* KS438 with a tolerance of up to 1.25 % (Nielsen et al., 2009; Kataoka et al., 2011; Vasylkivska and Patakova, 2020), *Leuconostoc pseudomesenteroides* IMAU70090, *Pediococcus acidilactici* IMAU20068, and *Enterococcus casseliflavus* IMAU10148 with a tolerance range of 3.25 – 3.5 % (Li et al., 2010; Vasylkivska and Patakova, 2020), *Staphylococcus sciuri* KM16 with a tolerance of up to 2.25 % (Goyal et al., 2019), and *Pseudomonas putida* with a tolerance of up to 6 % (Rühl et al., 2009; Vasylkivska and Patakova, 2020).

Enhancing butanol concentration is a key focus in biobutanol production (Li et al., 2020; Lin et al., 2023). Genetic engineering has succeeded in increasing the levels of butanol produced (Nawab et al., 2020). For example, upon inactivating the acetone decarboxylase gene (*adc*), *C. acetobutylicum* ATCC 55,025 and HKKO showed a notable reduction in acetone levels and a significant increase in the butanol/ABE ratio, achieving 11 g/L of butanol production. Moreover, overexpression of the acetaldehyde/alcohol dehydrogenase gene (*adhE2*) further elevated butanol output, resulting in hyper-butanol-producing strains with improved productivity, yielding up to 19.7 g/L of butanol (Du et al., 2022). Additionally, removing histidine kinase effectively enhanced the performance of *C. acetobutylicum*, raising the butanol titer from 12.6 to 18.2 g/L (Xu et al., 2014). Furthermore, replacing the *cat1* gene with *adhE1*/*adhE2* from *C. tyrobutyricum* led to the production of 26.2 g/L of butanol through batch fermentation at low temperatures (Zhang et al., 2018b).

Additionally, algae have emerged as an alternative substrate for biobutanol production (Rana and Parul, 2018). According to Shanmugam et al. (2021), microalgae can increase their carbohydrate content through pathway engineering, diverting flow towards carbohydrate metabolism rather than lipid production, which results in biobutanol production. Moreover, utilizing co-culture for butanol productivity can be another viable option (Wu et al., 2019; Cui et al., 2021). Oliva-Rodriguez et al. (2019) reported that *C. acetobutylicum* ATCC 824 demonstrated high productivity, yielding 8.28 g/L of butanol when co-cultured with *B. subtilis* CDB 555 using *Agave lechuguilla* as the substrate. *B. subtilis* aids in oxygen consumption, allowing strains like *Clostridium* to thrive and produce butanol without the need for chemical additives and nitrogen gas flushing.

As reported by Sun et al. (2019), other microorganisms have been genetically engineered to synthesize new products from syngas, expanding the scope of syngas fermentation technologies and improving metabolite productivity. A novel gene encoding diol dehydratase, found in various *Clostridium* species, was successfully transferred to *E. coli*, enabling the conversion of propane-1,2-diol isomers. Genetically modified *C. ljungdahlii* has the potential to produce isoprene from syngas by incorporating genes encoding isoprene synthase and isopentenyl diphosphate isomerase. Additionally, recombinant *C. autoethanogenum* has been engineered to biosynthesize 3-hydroxypropionate, a precursor for the biodegradable polymer poly(3-hydroxypropionic acid), highlighting the potential of genetic manipulation in altering enzyme pathways for enhanced product distribution.

In the past two decades, extensive research has explored alternative fermentation and recovery techniques for biobutanol production, including immobilized bioreactors, cell recycling systems, and innovative recovery methods like adsorption and gas stripping (Rai et al., 2022). Advances in solvent removal during fermentation have also been achieved through techniques such as reverse osmosis and pervaporation (Gottumukkala et al., 2019). This comprehensive exploration highlights the need to streamline the butanol fermentation process and adopt cocultivation technology or alternative methods, facilitating fermentation under non-strict anaerobic conditions (Lin et al., 2023).

Despite these advancements, challenges persist in the choice of raw materials for butanol production from *Clostridium* (Rana and Parul, 2018). The selection of raw materials is pivotal in determining the overall efficiency and economic viability of the process (Cavelius et al., 2023). Furthermore, variations in raw material composition and impurities can impact the fermentation process and the quality of the end product (Kótai et al., 2013; Sumardiono et al., 2020).

## 6 Future directions, recommendations, and research gaps

The advancement in *Clostridium*-driven butanol fermentation has achieved significant milestones in strain development (Du et al., 2022), bioprocess optimization (Magalhaes et al., 2018; Sagar et al., 2023), exploration of cost-effective substrates (Dziergowska et al., 2022), product recovery (Qureshi et al., 2014), and bioprocess modulation (Liu et al., 2012; Karstens et al., 2021). However, the persistent challenges of low productivity and high production costs remain substantial hurdles for large-scale butanol production (Birgen et al., 2019; Li et al., 2020; Guo et al., 2022). Therefore, future biobutanol production should prioritize enhancing efficiency and sustainability while maintaining affordability to compete with fossil fuels (Rathour et al., 2018; Malik et al., 2024).

Currently, processing costs using lignocellulosic hydrolysate and microalgae are prohibitively high, mainly due to biomass pretreatment and the use of exogenous cellulases, which diminishes economic competitiveness (Liu et al., 2021b; Krishnamoorthy et al., 2022; Lin et al., 2023). Microalgae pretreatment techniques are still under development, with researchers striving to achieve more efficient lipid products (Onumaegbu et al., 2018; Krishnamoorthy et al., 2022; Udayan et al., 2022). Although disruptive cell methods are effective, they require substantial energy, necessitating careful consideration of pretreatment methods to improve efficiency (Lee et al., 2013; Krishnamoorthy et al., 2022; Rahman et al., 2022).

Moreover, despite the significant operational costs of biofuel production, its use offers potential environmentally friendly alternatives (Jeswani et al., 2020; Malik et al., 2024). However, the lipid yield variation from microalgae emphasizes the need for systematic evaluation to select the most suitable pretreatment methods (Krishnamoorthy et al., 2022). On the other hand, in lignocellulosic waste, lignin degradation poses a major challenge in utilizing all lignocellulosic components (Liu et al., 2021b; Mujtaba et al., 2023). However, microorganisms capable of lignin degradation present a potential solution (de Medeiros et al., 2022; Zhang et al., 2023).

New discoveries such as lytic polysaccharide monooxygenases (LPMOs) have improved lignocellulosic degradation efficiency, while a better understanding of hydrolase mechanisms will help optimize degradation conditions to enhance saccharification efficiency (Bertini et al., 2018). Enzyme loading coordination and degradation efficiency are key to overcoming economic barriers to commercial processing (Liu et al., 2021b). Bacteria such as *Clostridium* spp., *Ruminococcus* spp. (Chukwuma et al., 2021; Gharechahi et al., 2023), and filamentous fungi like *Trichoderma* spp. (Li et al., 2019; Zhang et al., 2019) play a vital role in producing the enzymes required for lignocellulosic degradation (Chukwuma et al., 2021). By understanding the interactions and synergies among microorganisms, the efficiency of lignocellulosic degradation processes can be enhanced for broader industrial applications (Liu et al., 2021b).

The development of consolidated bioprocessing (CBP) technology to convert lignocellulose into butanol has the potential to replace butanol production from non-renewable sources at a lower cost (Re and Mazzoli, 2023). Research needs to explore the potential of protoplast fusion techniques to improve enzymatic activity and biobutanol production from agricultural waste (Cui et al., 2014; Mohtasebi et al., 2019; Re and Mazzoli, 2023). Advances in CBP development are achieved through combining (hemi)cellulolytic features and butanol production within a single microorganism or using a consortium of engineered microbes (Re and Mazzoli, 2023). Developing microbial strains tolerant to butanol is necessary to overcome its toxicity and improve fermentation yields (Peabody and Kao., 2016; Lin et al., 2023). CBP utilization is estimated to reduce capital and operational costs by 40–77 % compared to alternative process technologies such as simultaneous saccharification and fermentation, solid-state fermentation, or simultaneous saccharification and co-fermentation (Re and Mazzoli, 2023).

There are also other promising approaches for increasing butanol production. These include regulating redox metabolism to enhance coenzyme availability, using redox mediators, and integrating bioelectrochemistry into bioreactors (Liu and Yu, 2020; Re and Mazzoli, 2023). However, challenges to overcome include increasing electron transfer efficiency (Chen et al., 2020), improving product selectivity, and reducing butanol toxicity to microbes (Li et al., 2020). Additionally, the cost balance from an energy-economic perspective must be considered when implementing these approaches, as they require additional energy sources (Liu and Yu., 2020). Indeed, interdisciplinary collaboration will facilitate large-scale butanol production from biocatalysis to bioelectrocatalysis.

Moreover, research on the use of microalgae and syngas as alternative biobutanol substrates remains limited, with most reported studies confined to laboratory-scale investigations without reflection or assessment of commercial-scale production to create value (Yeong et al., 2018). In gas fermentation, lignocellulosic biomass can be gasified into synthesis gas, which is then fermented into hydrocarbons by acetogenic organisms (Daniell et al., 2012; Fernandez-Naveira et al., 2017a; He et al., 2022b). Metabolic engineering techniques have been applied to these organisms, opening opportunities for energy-dense fuel and chemical production (Riaz et al., 2022; Palaniswamy et al., 2023). Gas fermentation offers advantages in using non-food biomass, raw material flexibility, and production efficiency (Daniell et al., 2012; Dhakal and Acharya., 2021). Furthermore, the process offers selectivity, resilience, and high development potential (Daniell et al., 2012; Re, 2023).

## 7 Conclusions

The use of underutilized substrates, such as agricultural waste, MSW, macroalgae, and microalgae, offers significant opportunities as alternative energy sources, particularly in biobutanol production. However, the importance of innovation and interdisciplinary collaboration in addressing large-scale production challenges cannot be overstated. Despite advancements in strain development, bioprocess optimization, and exploration of cost-effective substrates, the challenges of low productivity and high costs remain substantial. In the current scenario, research is focused on improving production efficiency and sustainability through integrated processing technology (CBP) and the use of more efficient microorganisms in lignocellulose degradation. Looking forward, the exploration of alternative materials such as microalgae and syngas, along with the development of metabolic engineering and bioelectrochemical techniques, holds great potential to reduce dependence on fossil fuels, enhance the economic competitiveness of biofuels, and accelerate the transition towards cleaner and more affordable renewable energy.

## Funding

This work was funded by Universitas Padjadjaran through Riset Percepatan Lektor Kepala (RPLK) grant number 1616/UN6.3.1/PT.00/2024, awarded to Febri Doni and Academic Leadership Grant (ALG) grant number 1510/UN6.3.1/PT.00/2024, awarded to Nia Rossiana.

## CRediT authorship contribution statement

**Devina Syifa Nabila:** Conceptualization, Writing – original draft, Writing – review & editing. **Rosamond Chan:** Conceptualization, Writing – original draft, Writing – review & editing. **Rizky Riscahya Pratama Syamsuri:** Writing – original draft, Writing – review & editing. **Puspita Nurlilasari:** Writing – review & editing. **Wan Abd Al Qadr Imad Wan-Mohtar:** Writing – review & editing. **Abdullah Bilal Ozturk:** Writing – review & editing. **Nia Rossiana:** Writing – review & editing, Project administration, Supervision, Funding acquisition. **Febri Doni:** Writing – review & editing, Project administration, Supervision, Funding acquisition.

## Declaration of competing interest

The authors declare that they have no known competing financial interests or personal relationships that could have appeared to influence the work reported in this paper.

## Data Availability

No data was used for the research described in the article.
